# Current Indications of Secondary Enucleation in Retinoblastoma Management: A Position Paper on Behalf of the European Retinoblastoma Group (EURbG)

**DOI:** 10.3390/cancers13143392

**Published:** 2021-07-06

**Authors:** Christina Stathopoulos, Livia Lumbroso-Le Rouic, Annette C. Moll, Manoj Parulekar, Philippe Maeder, François Doz, Helen Jenkinson, Maja Beck Popovic, Guillermo Chantada, Francis L. Munier

**Affiliations:** 1 Jules-Gonin Eye Hospital, Fondation Asile des Aveugles, University of Lausanne, 1003 Lausanne, Switzerland; francis.munier@fa2.ch; 2 Department of Ocular Oncology, Institut Curie, 75005 Paris, France; livia.lumbroso@curie.fr; 3 Department of Ophthalmology, Amsterdam UMC, Vrije Universiteit Amsterdam, Cancer Center Amsterdam, 1081 HV Amsterdam, The Netherlands; a.moll@amsterdamumc.nl; 4 Birmingham Children’s Hospital Eye Department, Birmingham Women’s and Children’s NHS Foundation Trust, Birmingham B4 6NH, UK; manoj.parulekar@nhs.net; 5 Department of Radiology, Centre Hospitalier Universitaire Vaudois, 1011 Lausanne, Switzerland; philippe.maeder@chuv.ch; 6 SIREDO Centre (Care, Innovation and Research in Pediatric, Adolescent and Young Adult Oncology), Institut Curie and University of Paris, 75005 Paris, France; francois.doz@curie.fr; 7 Department of Pediatric Oncology, Birmingham Women’s and Children’s NHS Foundation Trust, Birmingham B4 6NH, UK; hjenkinson1@nhs.net; 8 Pediatric Hematology-Oncology Unit, Centre Hospitalier Universitaire Vaudois, 1010 Lausanne, Switzerland; Maja.Beck-Popovic@chuv.ch; 9 Hospital Sant Joan de Deu, 08950 Barcelona, Spain; gchantada@yahoo.com

**Keywords:** retinoblastoma, secondary enucleation, metastasis, external beam irradiation, intravenous chemotherapy, intra-arterial chemotherapy, survival

## Abstract

**Simple Summary:**

Although secondary enucleation (SE) is the treatment of choice for retinoblastoma eyes that did not respond favorably to conservative therapies, clear criteria for its indication are, however, currently missing. In this position paper on behalf of the European Retinoblastoma Group (EURbG), we discuss the available literature on SE, including its influence on metastases rate and survival, and propose guidelines to assist decision-making to interrupt eye-preserving therapies depending on the availabilities of advanced diagnostic and therapeutic modalities. Absolute indications to SE may be restricted to eyes with refractory tumor activity resisting all salvage treatments or eyes under apparent tumor control but no visual potential and irreducible complications. In contrast, eyes with an obscured optic nerve head and/or ocular complications amenable to specific surgical or medical management can be considered relative indications, provided that appropriate follow-up can be implemented and that parents are fully aware of a residual risk.

**Abstract:**

Secondary enucleation (SE) puts an irreversible end to eye-preserving therapies, whenever their prolongation is expected to violate the presumed state of metastatic grace. At present, it must be acknowledged that clear criteria for SE are missing, leading to empiric and subjective indications commonly related to disease progression or relapse, disease persistence masking the optic nerve head or treatment-related complications obscuring the fundus view. This absence of evidence-based consensus regarding SE is explained by the continuously moving frontiers of the conservative management as a result of diagnostic and therapeutic advances, as well as by the lack of studies sufficiently powered to accurately stratify the risk of metastasis in conservatively treated patients. In this position paper of the European Retinoblastoma Group (EURbG), we give an overview of the progressive shift in the indications for SE over the past decades and propose guidelines to assist decision-making with respect to when SE becomes imperative or recommended, with corresponding absolute and relative SE indications. Further studies and validation of biologic markers correlated with the risk of metastasis are expected to set more precisely the frontiers of conservative management and thus consensual criteria for SE in the future.

## 1. Introduction

Despite the significant advances made in retinoblastoma management over the last decades, secondary enucleation (SE) is sometimes inevitable in order to preserve the patient from metastatic disease and death, and remains to date the treatment of choice for eyes that did not respond favorably to conservative strategies. Current indications to discontinue eye-preserving therapies include progressive/relapsing disease [1,2,3,4,5,6,7,8,9,10,11,12,13,14,15,16,17,18,19,20,21,22,23,24,25,26,27,28,29,30,31,32,33,34,35,36,37,38,39,40,41,42], persistent disease obscuring the optic nerve head, loss of fundus view (secondary to poor pupillary dilatation, intraocular hemorrhage and/or cataract) [2,5,17,18,40,41,43,44], neovascular complications [13,35,43,45], rhegmatogenous/tractional retinal detachment [29,40], painful blind eye [16] and/or phthisis bulbi [10,11,23]. Sometimes, although rarely, SE may also be preferred by the parents due to the burden of prolonged conservative treatment on the child and/or family [12,39], or encouraged by the medical team due to lack of parental compliance even if reasonable conservative alternatives can still be considered.

Although rarely performed for retinoblastoma groups A–C, various studies report SE rates for advanced retinoblastoma groups D–E ranging from 29% to 74% when treated with systemic chemotherapy [18,22,46,47,48] and 0 to 61% when treated with intra-arterial chemotherapy [23,30,34,38,49,50]. Contrary to primary enucleation, where the presence of one or more International Intraocular Retinoblastoma Classification (IIRC) group E features at diagnosis such as neovascular glaucoma, massive intravitreal hemorrhage or diffuse infiltrating tumor have been considered, at least until recently, an absolute indication for immediate enucleation [51], clear consensual criteria for SE have never been established. In the event of disease progression and/or complications, the crucial decision to stop conservative management is therefore left to the expertise of the multidisciplinary team in charge, with the need to balance the potential benefits of additional globe-salvage treatments against the risks of disease progression and metastasis as well as to ensure full parental understanding of the potential consequences of retaining an eye.

The need to establish criteria for SE indications in the retinoblastoma management in order to harmonize the management and improve the care of retinoblastoma patients was raised by the European Retinoblastoma Group [52], a reference network for retinoblastoma involving more than 80 international experts of 24 countries dealing with retinoblastoma, in the annual meeting of 2019. A group of experts specialized in ocular oncology, pediatric oncology and neuroradiology from five major European Centers (Switzerland, France, England, Spain and Holland) were identified by the committee to address this question and provide guidelines for SE criteria.

## 2. Methods

Medline, Pubmed and Google Scholar were searched for English language scientific literature reporting on SE in retinoblastoma to summarize the actual knowledge on the topic. All studies with a minimum of 15 retinoblastoma patients managed conservatively and reporting on SE from January 1970 to December 2020 as well studies reporting exclusively on SE in retinoblastoma were included. Studies were categorized into three distinct groups: patients with SE after external beam irradiation +/− focal therapies (Table 1), patients with SE after first line systemic chemotherapy +/− focal therapies (Table 2) and patients with SE after first line or salvage intraarterial chemotherapy +/− focal therapies (Table 3). Secondary enucleation rates and indications, clinico-pathological correlations of the enucleated eyes, adjuvant treatments, metastasis rates and overall survival if available were noted for each study. Series with fewer than 15 patients, case reports, non-English studies and studies on retinoblastoma management not reporting on SEs were excluded. When institutions published studies with similar cohorts, the ones with the larger ones and/or the ones with the more detailed information including longer follow-ups were chosen. Independent screening of the literature using the inclusion/exclusion criteria was performed by two of the authors (C.S., F.L.M.).

Based on the results of the above-described literature review, we first discuss indications and metastasis rate in line with the different eras of disease management and finally propose SE criteria and follow-up guidelines for characteristic clinical situations as a result of a consensus made on behalf of the European Retinoblastoma Group (EURbG) by 10 retinoblastoma experts in ocular oncology (C.S., L.L.-L.R., A.M., M.P., F.L.M.) pediatric oncology (F.D., H.J., G.C., M.B.P.) and neuroradiology (P.M.).

## 3. Results

### 3.1. Shift of SE Indications over the Years

Since the beginning of conservative retinoblastoma management, the main reason to stop eye-preserving therapies has always been, and still is related, independently of the treatment modalities used, to uncontrolled tumor activity (80–90% of SE) [2,4,5,6,7,10,11,13,14,15,16,17,18,19,20,21,22,23,24,26,27,29,30,31,32,34,35,37,38,40,41,53,54,55,56], while the occurrence of intraocular complications, especially those obscuring the fundus view, explains the remaining cases (10–20% of SE) [2,5,10,11,13,16,17,18,21,23,28,29,34,39,40,41] (see Table 1, Table 2 and Table 3). Constant innovations in diagnostic and therapeutic retinoblastoma care have, however, allowed progressive improvement of tumor control and successful treatment of various complications that would otherwise compromise globe-salvage, leading, over the years, to a continuous shift in SE indications and a significant decrease of the overall SE rate [57].

### 3.2. The Role of Intra-Arterial, Intravitreal and Intracameral Chemotherapy and Management of Treatment-or Tumor-Related Complications

The greatest advance in the eye-preserving treatment of retinoblastoma has undeniably been brought about by the development of various techniques to safely deliver high chemotherapy drug concentrations into the different eye compartments, allowing an unprecedented control of both solid tumors and seeding [57]. First of all, the modern approach of super-selective intra-arterial chemotherapy introduced in 2008 [58] enabled not only to salvage heavily pre-treated eyes that would otherwise have faced SE, but also to achieve a higher control of retinal tumor (92% versus 62%, *p* = 0.002) and subretinal seeding (86% versus 31%, *p* = 0.006), compared to intravenous chemotherapy for advanced treatment-naïve retinoblastoma groups D and E [59]. Furthermore, intra-arterial chemotherapy was also shown to be effective in isolated cases of massive choroidal [60,61] and iris invasion [62], but not in ciliary body invasion requiring brachytherapy for tumor control [57,63], with no reported metastasis nor deaths, indicating that posterior and anterior uveal involvement should not necessarily be considered as an absolute criterion for enucleation, nor a definite indication for adjuvant chemotherapy [64,65,66]. In 2012, the introduction of a safety-enhanced technique to perform intravitreal injection in an eye with active tumor [67] permitted almost absolute control (close to 100% of the cases) of the vitreous disease [43,57,68,69,70,71,72], previously leading to SE in about 50% of the eyes managed with first line external beam irradiation or chemoreduction [9,46] and 36% of those managed with first line intra-arterial chemotherapy [73]. Noteworthy, both intravitreal [74] and/or intra-arterial injections [75,76] can be successfully repeated in relapsing cases. In addition to its use for active tumor treatment, intravitreal melphalan has also enabled a more secure management of various complications, necessitating intraocular surgery such as cataract [57] or tractional retinal detachment [77,78,79]. Similarly, the use of intravitreal anti-VEGF injections performed according to the same technique as intravitreal chemotherapy has permitted the eye-preserving management of treatment- or tumor-related neovascular complications, earlier commonly treated with immediate enucleation [80]. Finally, the more recent inception in 2015 of a technique adapted to inject safely into the anterior and posterior chambers, namely intracameral chemotherapy [81,82], has shown promising results for the control of aqueous seeding [57,83,84], previously also treated with immediate enucleation.

While the use of intra-arterial, intravitreal and intracameral chemotherapies is considered safe, with no reported life-threatening related adverse effects [62,85,86], their wide implementation in retinoblastoma management has raised concerns on the possible negative consequences of a lower systemic chemotherapy exposure and its potential benefit in preventing systemic metastasis in children with microscopically-undetected disseminated disease who relapse with metastatic disease following completion of treatment [87,88]. Although more studies with longer follow-ups are needed to be able to reach a conclusion, such fears have not, however, been confirmed to date. Indeed, when considering the studies reporting on metastatic disease, a similar metastasis rate of about 2% is found in children treated with systemic chemotherapy [15,16,19,22,25,26] or intra-arterial chemotherapy [23,29,33,34,35,37,38,42], whereas, according to a retrospective multicentric survey including more than 1100 patients managed with primary (*n* = 464) or salvage intra-arterial chemotherapy (*n* = 713) over a 10-year period, the risk of metastatic death from retinoblastoma has been estimated to be less than 1% [89].

### 3.3. The Role of Ancillary Testing

Along with the advances brought about by the emergence of new treatment modalities, the development of various imaging techniques aiming at evaluating the disease extent (especially if there is fear of exteriorization or in case of fundus view loss) as well as the presence or not of tumor activity has been crucial to set up the limits of eye-preserving treatment. Thus, spectral domain optical coherence tomography (OCT) has been instrumental in the eye-preserving management of cases with choroidal [60] or epipapillary relapse [90] by allowing early tumor detection and close monitoring of the treatment response. Recently, anterior segment OCT has also been reported to accurately detect in vivo tumor progression into the Schlemm’s canal [91]. Fluorescein angiography allows to assess the tumoral and retinal vascularization status [92] and to monitor the treatment response to intraocular vascular complications [80]. In the case of opaque media, ultrasonography (B-scan) provides useful information to assess any tumor growth or optic nerve threat, while high-resolution contrast-enhanced magnetic resonance imaging (MRI) enables the evaluation of tumor activity and potential exteriorization. While its sensitivity and specificity in detecting scleral and peribulbar invasion is considered to be near 100%, MRI is, however, less sensitive to detecting early choroidal [93] or postlaminar optic nerve invasion [93,94]. Finally ultrasonic biomicroscopy proved to be instrumental, not only for the determination of a tumor-free entry meridian prior to intravitreal injection for vitreous disease in case of compromised pupil dilation or presence of opaque media [57,71], but also for the assessment and monitoring of tumor invasion of the ciliary body and/or posterior chamber [57,81].

### 3.4. Influence of Delayed Enucleation on Metastasis Rate and Survival

The advent of the afromentioned diagnostic and therapeutic modalities resulted in more advanced diseased eyes and more heavily pretreated ones escaping primary and SE respectively, raising concerns of a potential negative impact on metastasis rate and overall survival in case of delayed enucleation [87]. In a series of 45 group E eyes enucleated for persistent disease after first line systemic chemotherapy, the authors reported that SE delayed for more than three months after diagnosis was associated with mortality in four patients as a result of pathologic downstaging of the disease and reduced surveillance leading to inappropriate management of unrecognized high-risk factor for metastasis [19]. In two studies comparing histopathology in eyes treated with first line enucleation versus SE, others demonstrated, however, that prolonged times to enucleation were associated with the presence of high-risk features but not to the development of metastasis nor mortality [21,24], suggesting that prompt recognition of refractory disease followed by timely enucleation and adjuvant therapy for high risk factors can efficiently prevent metastatic dissemination [21]. Interestingly, in a study including 24 eyes enucleated after chemoreduction at an average time of five months after loss of fundus view, 22 (92%) had viable tumor cells on histopathology, but none of them showed high-risk features [55]. Finally, in two other studies comparing advanced retinoblastoma group D/E treated with either first line systemic chemotherapy or first line intra-arterial chemotherapy versus primary enucleation, conservative treatment was not found to increase the risk of orbital recurrences, metastatic disease or death [22,36].

### 3.5. Indication for SE and Management of High-Risk Pathologic Factors

The establishment of clear guidelines regarding the optimal timing of SE and the need for post-enucleation adjuvant chemotherapy is hindered by the present lack of studies having focused on that subject with only little information available from the studies reporting their treatment outcomes (especially regarding retention times or management of cases with loss of fundus view, and clinicopathologic correlations) and the overall low rates of metastatic disease. On the other hand, the absence of a consensus for the definition of high-risk pathologic factors, with some considering anterior chamber invasion or isolated massive choroidal invasion as a high-risk features for metastasis [95], while others not [64,96,97], as well as considerable variations in the use and type of post-enucleation adjuvant therapies precludes any conclusions regarding the metastasis risk and comparison of survival rates [95]. The use of the recently-proposed classification of retinoblastoma at relapse (RSU classification), which aims to standardize the treatment for relapse based on the recurrence localization [57], and the classification of regressed retinoblastoma (RB-Recist) [98] should allow a better comparison of treatment outcomes and help define SE criteria in the coming years. Last but not least, the future validation of tumor-specific biomarkers in liquid biopsies may revolutionize the conservative retinoblastoma management by stratifying the risk of metastasis in a histopathologic-independent manner and/or diagnosing minimally disseminated disease in blood, cerebrospinal fluid or bone marrow [99].

In the expectation of prospective studies that could bring evidence-based answers to the above concerns, on behalf of the European Retinoblastoma Group (EURbG), we propose guidelines to assist decision making with respect to when SE becomes imperative (absolute indication) or recommended (relative indication for SE) as a result of a consensus based on the clinical experience of each co-author active in European referral center and the above discussed review of the literature (see Table 4).

## 4. Conclusions

Despite a growing use of conservative treatments, SE still has a central role to play in the management of retinoblastoma to preserve the patient from metastasis and death. Although the need for SE cannot, to date, be unequivocally delineated, it is however possible to draw distinction borders between absolute and relative indications for SE depending on the available diagnostic and therapeutic modalities and on consensus among the local multidisciplinary retinoblastoma team (Table 4). Thus, absolute indications may be restricted to eyes with refractory tumor activity resisting all therapeutic modalities or eyes under apparent tumor control but no visual potential and untreatable intra-ocular complications. In contrast, eyes with an obscured optic nerve head and/or ocular complications amenable to specific surgical or medical management can be considered relative indications to SE or may be conditionally maintained, provided that appropriate follow-up can be implemented and that parents are fully aware of a residual risk.

## Figures and Tables

**Table 1:** Secondary enucleations (SE), orbital invasion, metastases and mortality rates after first-line external beam radiation therapy (EBRT): literature review.

Author (Year)	Study Years	Eyes/Patients		Classification Group		Treatments †		Indication for SE *n* (% SE Eyes)	Mean/Median Retention Time, Months (Range)	HRF, *n* (% SE Eyes)	Adjuvant IVC, *n* (% SE Eyes with HRF)	Mean/Median Follow-Up, Months (Range)	Orbit Invasion, *n* (% Patients)	Metastases, *n* (% Patients)	Deaths, *n* (% Patients)
		All	SE Eyes	All	SE Eyes	All	SE Eyes ^††^								
Thompson et al. (1972) [1]	1956–1970	34/33	9/9	RE: I = 17 II = 3 III = 5 IV = 4 V = 5	RE: I = 1 II = 2 III = 2 IV = 2 V = 2	EBRT = 34	EBRT = 9	Active disease = 9 (100) *	17 (4–43)	na	na	na	na	1 (3) ^a^	1 (3) ^a^
Egbert et al. (1978) [2]	1956–1974	38	16	RE: I = 8 II = 5 III = 8 IV = 2 V = 15	RE: I = 1 II = 2 III = 1 IV = 2 V = 10	EBRT = 38	EBRT = 16	Active disease = 7 (44) * Blind eye = 8 (50) - 2ry NVG = 5 - 2ry VH = 1 - 2ry RD = 1 - 2ry cataract = 1 Unknown = 1 (6)	(0–4 years)	na	na	10 years (2.5–21 years)	na	na	0 (0)
Foote et al. (1989) [3]	1977–1987	25/18	5/na	RE: I = 1 II = 1 III = 13 IV = 4 V = 6	RE: III = 2 IV = 2 V = 1	EBRT = 25	EBRT = 5	Active disease = 5 (100) *	10 (2–25)	na	na	32 (7–113)	na	na	0 (0)
Toma et al. (1995) [4]	1986–1992	67/53	5/na	RE: I = 18 II = 33 III = 11 IV = 5	RE: II = 4 III = 1	EBRT = 67	EBRT = 5	Active disease = 5 (100) *	na	na	na	35 (12–82)	na	na	na
Hernandez et al. (1996) [5]	1980–1991	34/27	9/na	RE: I–II = 14 III = 7 IV–V = 13	na	EBRT = 27 Plaque = some	EBRT = 9 Plaque = 2	Active disease = 7 (67) * Irradiation complications = 2 (33) - Glaucoma = 1 - NVX + hemorrhage = 1	na	na	na	35 (12–93)	0 (0)	0 (0)	0 (0)
Blach et al. (1996) [6]	1979–1991	180/123	32/na	RE: I = 41 II = 32 III = 22 IV = 13 V = 72	RE: I-III = 8 IV/V = 24	EBRT = 182	EBRT = 32	Active disease = 32 (100)*	na	na	27 (84) ^b^	(4–159)	na	6 (5)^c^	14 (11)^c^
Merchant et al. ocular (2002) [7]	1978–1998	49/38	6/na	RE: I = 14 II = 10 III = 11 IV = 2 V = 7 na = 5	na	EBRT = 49 IVC = 10	EBRT = 6	Active disease = 6 (100) *	15 (7–68)	na	na	89 (34–373)	1 (3) ^d^	0 (0)	4 (11) ^d^
Phillips et al. (2003) [8]	1965–1997	47/35	13	RE: I–II = 16 III = 7 IV–V = 20 na = 4	RE: I–II = 1 III = 1 IV = 1 V = 8 na = 2	EBRT = 47 IVC = 9 ^e^	EBRT = 13	na	within 2 years post EBRT	na	na	na	na	1 (3) ^f^	5 (14) ^f^
Abramson et al. (2004) [9]	1979–2002	63/53	25/na	RE: Vb = 63	RE: Vb = 25	EBRT = 63	EBRT = 25	Active disease = 25 (100) *	14 (5–107)	na	na	na	2 (4) ^g^	3 (6)	5 (9) ^h^
Choi et al. (2010) [10]	1987–1998	32/25	8/7	RE: II = 5 III = 16 IV = 4 V = 7	RE: II = 1 III = 3 IV = 2 V = 2	EBRT = 32 IVC = 22 ^i^	EBRT = 8	Active disease = 7 (88) * Cataract + phthisis = 1 (12)	na	na	na	150 (55–249)	na	2 (8)	2 (8) ^j^
Camp et al. (2019) [11]	1980–1994	48/26	4/na	na	na	EBRT = 24 Plaque/FT = 24	EBRT era ^k^	Active disease = 2 (50) * NVG = 1 (25) Phthisis = 1 (25)	na	na	na	125 (4–330)	na	1(4) ^l^	5 (19) ^l^

Legends: ^†^= some of the eyes received additional focal treatments; ^††^ = pre-enucleation treatments; HRF = histopathological risk factors for metastasis; IVC = intravenous chemotherapy; * =active disease include persistent, progressive and/or relapsing disease; na = non-available; RE = Reese–Ellsworth Classification; NVG = neovascular glaucoma; VH = vitreous hemorrhage; RD = retinal detachment; NVX = neovascularization; FT = focal treatments. ^a^ = one patient died of central nervous system metastases; ^b^ = those patients received chemotherapy for evidence of extraocular disease at time of SE; ^c^ = six children died of metastatic disease, 7 of pinealoblastoma and 1 of a secondar neoplasm within the radiation field; ^d^ = one child died of orbital metastasis, 1 of pinealoblastoma and 2 of osteosarcoma; ^e^ = nine children received chemotherapy after enucleation of a higher stage contralateral eye; ^f^ = one patient died of metastatic retinoblastoma, 3 of second malignancies and 1 death was accidental; ^g^ = two patients developed orbital recurrence in the contralateral eye that had been treated with primary enucleation.; ^h^ = all died of metastatic retinoblastoma; ^i^ = chemotherapy was given for high stage contralateral tumors that had been enucleated to control microscopic tumor; ^j^ = two patients died after involvement of central nervous system; ^k^ = some of them may have been treated with plaque brachytherapy or focal treatments only; ^l^ = one patient died of metastasis, 2 of second malignancies, 1 of pinealoblastoma and 1 of respiratory failure.

**Table 2:** Secondary enucleations (SE), orbital invasion, metastases and mortality rates after first-line intravenous chemotherapy (IVC): literature review.

Author (Year)	Study Years	Eyes/Patients		Classification Group		Treatments †		Indication for SE *n* (% SE Eyes)	Mean/Median Retention Time, Months (Range)	HRF, *n* (% SE Eyes)	Adjuvant IVC, *n* (%SE Eyes with HRF)	Mean/Median Follow-Up, Months (Range)	Orbit Invasion, *n* (% Patients)	Metastases, *n* (% Patients)	Deaths, *n* (% Patients)
		All	SE Eyes	All	SE Eyes	All	SE Eyes ^††^								
Nenadov Beck et al. (2000) [12]	1995–1998	33/24	6/5	RE: I = 5 II = 10 III = 3 IV = 1 V = 14	RE: V = 5	IVC = 33 EBRT = 13	IVC = 6 EBRT = 2	Active disease = 5 (83) Parental wish = 1 (17)	(0–8)	na	na	31 (4–41)	0 (0)	1 (4) ^a^	1 (4) ^a^
Rodriguez-Galindo et al. (2003) [13]	1996–2000	43/25	13/11	RE: I = 7 II = 12 III = 5 IV = 3 V = 16	RE: II = 2 III = 2 IV = 3 V = 6	IVC = 43 EBRT = 19	IVC = 13 EBRT = 9	Active disease = 10 (77) * NVG = 3 (23)	(14; 8–33)	3 (23) ^b^	3 (100)	32 (10–65)	na	na	0 (0)
Gündüz et al. (2004) [14]	1998–2003	105/71	32/na	RE: I = 4 II = 23 III = 7 IV = 35 V = 36	RE: II = 1 III = 1 IV = 7 V = 23	IVC = 105 EBRT = 26 Plaque = 8	IVC = 32 EBRT = 6	Active disease = 32 (100) *	na	na	0	26 (6–49)	na	na	1 (1) ^c^
Schiavetti et al. (2005) [15]	1992–na	58/46	21/na	RE: I = 10 II = 16 III = 9 IV = 6 V = 17	RE: I = 1 II = 4 III = 3 IV = 2 V = 11	IVC = 58 EBRT = 10	IVC = 21 EBRT = 3	Active disease = 21 (100) *	na	na	na	53 (11–125)	na	1 (2) ^d^	4 (9) ^d^
Chantada et al. (2007) [16]	1995–2002	-	139/122	-	RE: I–IV = 25 V = 91 na = 23	-	IVC = 139 EBRT = 35	Active disease = 136 (98) * Painful blind eye = 3 (2) ^e^	(10; 1–90) ^f^	41(29) ^g^	8 (20) ^h^	(54; 12–97)	2 (2) ^i^	3 (2) ^i,j^	2 (2) ^i,j^
Lumbroso-Le Rouic et al. (2008) [17]	1998–2002	115/83	23/20	IIRC: A = 19 B = 48 C = 19 D = 29	IIRC: B = 2 C = 4 D = 17	IVC = 115 EBRT = 13	IVC = 23 EBRT = 7	Active disease = 18 (78) * No fundus view = 5 (22) - 2ry to massive VH = 5	na	na	na	51 (32–72)	na	na	na
Shin et al. (2010) [18]	1997–2007	65/52	31/na	IIRC: A = 8 B = 14 D = 42 E = 1	IIRC: B = 4 D = 27	IVC = 65	IVC = 31	Active disease = 29 (94) * No fundus view = 2 (6) - 2ry hyphema and VH = 1 - 2ry cataract =1	na	na	na	54 (7–115)	na	na	1 (2) ^k^
Zhao et al. (2011) [19]	2006–2008	-	55/55	-	IIRC: D = 10 E = 45	-	IVC = 55	Active disease = 55 (100) *	3 (0.2–19)	7 (13) ^l^	some	25 (12–38)	na	5 (9)	5 (9) ^m^
Bartuma et al. (2014) [20]	2001–2011	46/24	13/12	IIRC: A = 8 B = 25 C = 1 D = 11 E = 1	IIRC: B = 3 C = 1 D = 7 E = 1	IVC = 46 EBRT = 4	IVC = 12 EBRT =3	Active disease = 12 (100) * - VS and/or SRS = 5 - large retinal tumor = 3 - growth on the ON = 3 - large tumor and extensive RD = 2	14 (1–43)	3 (25) ^n^	0 (0)	60 (13–144)	0 (0)	0 (0)	0 (0)
Brennan et al. (2015) [21]	1997–2013	-	63/60	-	IIRC: B = 13 C = 7 D = 34 E = 3 na = 6	-	IVC = 62 IAC = 1 EBRT = 26 Plaque = 10	Active disease = 56 (89) * VS = 40 SRS = 21 RD = 39 Other = 7 (11) ^o^	19 (1–13 years) With HRF: 30 (1–157) With no HRF: 16 (1–72) *p* = 0.018	13 (21) ^p^	10 (77) ^q^	0 (0)	0 (0)	0 (0)	0 (0)
Berry et al. (2017) [22]	1995–2015	139/na	58/na	IIRC: D = 102 E = 37	IIRC: D = 30 E = 28	IVC = 139 EBRT = 39	IVC ± EBRT = 58	Active disease = 58 (100) *	14 (8;1–118)	0 (0)	0 (0)	86	1 (1)	1 (1) ^r^	1 (1) ^r^
Munier et al. (2017) [23]	1997–2008	23/23	10/10	IIRC: D = 23	IIRC: D = 10	IVC = 23 Plaque = 2	IVC ± plaque = 10	Active disease = 9 (90) * seeding ± RD = 9 Phthisis bulbi = 1 (10)	17 (1–48)	2 (20)	2 (100)	105 (29–218)	0 (0)	0 (0)	0 (0)
Fabian et al. (2017) [24]	2002–2014	-	24/na	-	IIRC: D = 24	-	IVC = 24 IAC = 11 plaque = 5 EBRT = 5 IvitC = 4 ^s^	Active disease = 5 (21) * - VS = 2 - AC = 2 - Vitreous base relapse + diffuse subretinal hemorrhage = 1 na = 19 (79)	With HRF: 27 (17–40) ^s^ Without HRF: 9 (3–91) ^s^	5 (21) ^t^	5 (100)	72 (14–153) ^u^	0 (0)	0 (0)	0 (0)
Shields et al. (2020) [25]	1994–2019	964/554	161/na	ICRB: A = 54 B = 200 C = 128 D = 224 E = 225 na = 123	ICRB: A = 1 B = 9 C = 4 D = 41 E = 82 na = 24	IVC ± IAC ± plaque ± EBRT ± IvitC = 964	IVC ± IAC ± plaque ± EBRT ± IvitC = 161	na	15 (10, 1–191)	na	na	77 (0–299) ^v^	na	11 (2)	7 (1) ^w^
Gündüz et al. (2020) [26]	1998–2018	276/na	75/na	ICRB: A = 22 B = 114 C = 28 D = 90 E = 97	ICRB: *B = 16* *C = 4* *D = 49 na* *E = 81*	IVC = 254 IAC = 3 FT = 19 ±EBRT ±Plaque ±salvage IAC	IVC ± EBRT ± IAC Plaque = 9	Active disease = 75 (100) *	na	10 (13)	10 (100)	77 (1–268)	na	1 (na) ^x^	2 (na) ^x^
Alkatan et al. (2020) [27]	2013–2017	-	28/26	-	ICRB: D + E = 28	-	IVC = 27 EBRT = 14	Active disease = 28 (100) *	8 (0–38)	6 (21)	6 (21) ^y^	na	1 (4) ^z^	3 (12) ^z^	3 (12) ^z^

Legends: ^†^ = potential additional focal treatments are not mentioned; ^††^ = pre-enucleation treatments; HRF = histopathological risk factors for metastasis; na = not available; CTT = chemothermotherapy; EBRT = external beam irradiation; * = active disease include persistent, progressive and/or relapsing disease; VS = vitreous seeds; SRS = subretinal seeds; NVG = neovascular glaucoma; IIRC = International Intraocular Retinoblastoma Classification; ON = optic nerve; RD = retinal detachment; VH = vitreous hemorrhage; pTNM = pathological Tumor Node Metastasis Classification; IvitC = intravitreal chemotherapy; IAC = intraarterial chemotherapy; Carbo = carboplatin; FT = focal treatment; ^a^ = 1 patient died of progressive disease despite bilateral EBR and ultimately bilateral enucleation; ^b^ = deep choroidal invasion and/or extension into the ciliary body; ^c^ = 1 patients died of pinealoblastoma; ^d^ = 1 patient died of heart malformation, 2 because parents refused advised enucleation at relapse and 1 of brain metastasis despite high-dose chemotherapy; ^e^ = two of them had active tumor but no HRF; ^f^ = median retention time was 18 months (range 0.2–67) for the eyes with HRF and 10 months (range 0.4–114) for those without HRF, (*p* = 0.06); ^g^ = among those eyes, 39 had choroidal invasion which was massive in 19 with an additional intrascleral invasion in 6 eyes and transscleral invasion in 3 others. Six eyes had anterior segment invasion, with the latter being the only HRF in one case. Scleral involvement in 9 eyes was concomitant to post-laminar optic nerve with subarachnoid extension in 2 cases. Some patients had incomplete pathological reports; ^h^ = eight patients received adjuvant therapy because of scleral invasion, combined with post-laminar optic nerve with subarachnoid invasion in 2 cases. One case with intrascleral invasion did not receive adjuvant treatment because of parental decision. Four cases (two with subarachnoid invasion) received also orbital radiotherapy. One of them was treated with orbital exenteration after an orbital relapse; ^i^ = one patient with primary enucleation of one eye with no HRF developed orbital invasion while receiving adjuvant chemotherapy after SE of the contralateral eye showing scleral invasion. The child died of CNS relapse despite orbital exenteration. Another developed orbital relapse, 9 months after SE with no HRF treated with chemotherapy and autologous stem cell rescue and has remained disease-free for 54 months; ^j^ = one patient developed bone-metastasis 32 months after SE with no HRF. The child was treated with high dose chemotherapy, autologous stem cell and bone-irradiation and remained disease-free at a 20-month follow-up. Another developed CNS relapse after delayed SE (4 months) of the only remaining eye due to parent’s refusal, showing extrascleral invasion for which he received adjuvant chemotherapy. The child achieved a second complete remission with chemotherapy, radiotherapy and high dose chemotherapy 1 year after the enucleation but finally died of a subsequent leptomeningeal relapse; ^k^ = one patient died due to Fanconi syndrome; ^l^ = 20 eyes were stage pT1, 28 were pT2 and 7 were pT3/T4 according the American Joint Committee on Cancer pTNM classification (Finger PT, Harbour JW, Murphree AL et al. Retinoblastoma, in Edge SB, Byrd DR, Carducci MA, et al. (eds). AJCC Cancer Staging Manual, New York, NY, Springer, 2010, pp. 561–568); ^m^ = all died subsequently to CNS metastasis after SE of a Group E eye at an interval of more than 3 months from retinoblastoma diagnosis. All four that had HRF had received adjuvant chemotherapy. Specifically, two had tumor at the cut end of optic nerve (one with concomitant massive choroidal invasion) but died despite 6 and 7 cycles of adjuvant chemotherapy, respectively. One had tumor past lamina cribrosa but died of brain and spinal metastasis despite adjuvant and intrathecal chemotherapy. One had massive choroidal and scleral invasion and died after three cycles of adjuvant chemotherapy. One child had no HRF on the enucleated eye but died after further treatment refusal for the contralateral eye.; ^n^ = including choroidal infiltration (*n* = 3) and anterior chamber growth (*n* = 2); ^o^ = including neovascular glaucoma, poor visual potential, pain and/or vitreous hemorrhage; ^p^ = 13 eyes displayed HRF including anterior chamber invasion (*n* = 3), ciliary body invasion (*n* = 8), massive choroidal invasion (*n* = 4), postlaminar optic nerve invasion (*n* = 1), scleral invasion (*n* = 7) and extraocular disease (*n* = 2); ^q^ = 3 patients had additional adjuvant EBRT. Three patients with HRF did not receive adjuvant therapy but none of these experienced recurrence or metastasis; ^r^ = one patient with metastatic disease died; ^s^ = correspond to a cohort of 24 SE eyes after first line IVC ± salvage IAC. Retention times between the eyes with HRF and those without HRF were not significantly different *(p = 0.729)*; ^t^ = two eyes had ciliary body, iris ± anterior chamber involvement and 3 had massive choroidal invasion ± anterior chamber ± iris and ciliary muscle involvement; ^u^ = correspond to a cohort of 64 patients; ^v^ = mean follow-up in a total of 869 eyes in 540 patients; ^w^ = 4 patients died of pinealoblastoma, 2 of metastasis and one of a stroke following glioblastoma resection; ^x^ = one patient with retrolaminar optic nerve invasion at histopathological examination died after developing CNS metastasis. Another patient that had superficial scleral invasion on SE died of pinealoblastoma; ^y^ = 3 eyes were stage pT2b and 3 were stage pT3a according the 7th American Joint Committee on Cancer pTNM classification; ^z^ = 1 patient with bilateral SE died after developing orbital and CNS metastasis; 2 other patients died from metastatic disease: one patient with bilateral SE that developed hepatic and retroperitoneal extensive metastasis and one with SE of one eye and progressive disease of the remaining eye whose parents refuses SE. All the three patients who died had at least one secondary enucleated eye with HRF.

**Table 3:** Secondary enucleations (SE), orbital invasion, metastases and mortality rates after first line (1°) or salvage intra-arterial (2°) chemotherapy (IAC): literature review.

Author (Year)	Study Years	Eyes/Patients		Classification Group		Treatments †		Indication for SE, *n* (% SE Eyes)	Mean Retention Time, Months (Range)	HRF, *n* (% SE Eyes)	Adjuvant IVC, *n* (% SE Eyes with HRF)	Mean/Median Follow-Up, Months (Range)	Orbit Invasion, *n* (% Patients)	Metastases, *n* (% Patients)	Deaths, *n* (% Patients)
		All	SE Eyes	All	SE Eyes	All	SE Eyes *								
Abramson et al. (2012) [28]	2006–2011	30/na	1/1	COG: B = 19 C = 11	COG: C = 1	na = 29 2° = 1	IAC: 2° = 1	2nd IAC not feasible for technical reason = 1 (100)	na	na	na	16 (3–33)	0 (0)	0 (0)	0 (0)
Palioura et al. (2012) [29]	2006–2010	37/34	5/5	COG: D = 31 E = 6	na	IAC: 1° = 27 2° = 10 IVC = 9 EBRT = 3	IAC: 1° = 3 2° = 2 IVC = 2 EBRT = 1	Active tumor = 4 (80) Tractional RD = 1 (20)	na ^a^	na	na	21 (1–42)	0 (0)	1 (3) ^b^	0 (0)
Thampi et al. (2013) [30]	2010–2012	20/16	6/6	ICRB: A = 1 B = 4 C = 2 D = 11 E = 2	ICRB: D = 5 E = 1	IAC: 1° = 12 2° = 8 IVC = 8	IAC 1° = 5 IAC 2° = 1 EBRT = 1 IVC = 2	Active tumor = 6 (100) -VH obscuring fundus view = 2	na	1 (17) ^c^	2 (100) ^d^	All: 15 (1–29) SE group: 15 (6–29)	0 (0)	0 (0)	0 (0)
Shields et al. (2013) [31]	2008–2011	14/14	6/6	ICRB: D = 6 E = 8	ICRB: D = 2 E = 4	IAC: 2° = 14 IVC = 14	IAC: 2° = 6	Active tumor = 6 (100) - RT = 2 - SRS +/− VS = 3 - suspicion ON invasion on MRI = 1	na	0 (0)	0 (0)	All: 24 (17, 5–48) SE group: 19 (9–36)	0 (0)	0 (0)	0 (0)
Venturi et al. (2013) [32]	2008–2010	39/36	8/8	TNM: 1a = 1 1b = 12 2a = 8 2b = 14 3a = 4	RE: Vb = 8	IAC: 1° = 17 2° = 22 IVC = 21 EBRT = 1 Plaque = 1 IvitC = 1	IAC: 1° = 7 2° = 1	Active tumor = 8 (100)	na	pT1 = 6 (75) pT3a = 1 (12) na = 1 (12) ^e^	na	13 (1–27) ^f^	0 (0) ^f^	0 (0) ^f^	0 (0) ^f^
Bracco et al. (2013) [33]	2008–na	52/47	18/na	IIRC: A = 5 B = 18 C = 4 D = 25	IIRC: D = 12 na = 6	IAC: 1° = 22 2° = 30 IVC = 30 EBRT = 1 Plaque = 1 IvitC = 1	IAC: 1° = 12 2° = 6	na	na	na	na	40 (19–61) ^f^	0 (0) ^f^	0 (0) ^f^	0 (0) ^f^
Shields et al. (2014) [34]	2009–2013	70/70	23/23	ICRB: B = 1 C = 4 D = 17 E = 14 na = 34	ICRB: D = 1 E = 9 na = 13	IAC: 1° = 36 2° = 34 IVC = 34	IAC: 1° = 10 2° = 13	After IAC1°: na = 10 After IAC2°: Active tumor = 10 (77) VH = 2 (15) NVG = 1 (8)	na	na	na	19 (na)	0 (0)	0 (0)	0 (0)
Ong et al. (2015) [35]	2010–2013	17/12	7/6	ICRB: B = 3 C = 1 D = 1 E = 12	ICRB: B = 1 E = 6	IAC: 1° = 6 2° = 11 IVC = 11 EBRT = 4 IvitC = 3	IAC: 1° = 2 2° = 5 EBRT = 1	Active tumor = 5 (71) - RT + VS ± VH ± RD = 3 - VS + VH = 1 - NVG + corneal haze = 1 VH = 2 (29)	na	3 (43) ^g^	3 (100) ^g^	All: 21 (5–43) SE group: 22 (12–35)	0 (0) ^g^	3 (25) ^g^	2 (17) ^g^
Yannuzzi et al. (2015) [36]	2006–2014	77/72	10/na	COG: C = 2 D = 52 E = 23	na	IAC: 1° = 77	IAC: 1° = 10	na	na	1 (10) ^h^	na	39 (9–104)	1 (1) ^i^	3 (4) ^i^	0 (0) ^i^
Akyuz et al. (2015) [37]	2011–2014	56/46	19/na	ICRB: A = 7 B = 6 C = 16 D = 19 E = 8	na	IAC: 1° = 12 2° = 44 *IVC = 44*	IAC: 1° = 3 2° = 16	Active tumor =19 (100) - *VS = 10* - *na = 9*	na	6 (32) ^j^	6 (100) ^j^	12 (1–28)	1 (2) ^j^	1 (2) ^j^	2 (4) ^j^
Abramson et al. (2016) [38]	2006–2012	112/103	24/na	COG: D = 112	COG: D = 24	IAC: 1° = 54 IVC = 7 2° = 58 IVC = 51 EBRT = 15 Plaque= 4	IAC: 1° = 8 2° = 16 EBRT + IVC = 5 EBRT = 1 IVC = 9 Plaque = 1	na	na	na	na	34 (2–110)	0 (0)	3 (6) ^k^	1 (2) ^l^
Abramson et al. (2016) [39]	2008–2015	120/60	4/4	COG: A = 2 B = 18 C = 24 D = 56 E = 20	COG D = 2 E = 2	IAC: 1° = 30 2° = 30	IAC: 1° = 2 2° = 2	Active tumor = 3 (75) Parental choice = 1 (25)	na	na	na	na	0 (0)	0 (0)	1 (2) ^m^
Shields et al. (2016) [40]	2008–2015	66/66	18/18	ICRB: B = 3 C = 4 D = 36 E = 23	ICRB: D = 6 E = 12	IAC: 1° = 66	IAC: 1° = 18 IvitC =some	Active tumor = 10 (56) Other =8 (44) (VH, NVG and/or total RD)	(0.1–20)	na	na	(6–65)	0 (0)	0 (0)	0 (0)
Chen et al. (2017) [41]	2011–2013	107/73	23/na	IIRC: B = 11 C = 11 D = 56 E = 29	IIRC: D = 12 E = 11	IAC: 1° = 30 2° = 77 *IVC = 64*	IAC: 1° =9 2° = 13	Active tumor = 19 (83) - *RT = 12* - *VS = 4* - *SRS = 3* VH = 4 (17)	na	na	na	14 (3–28)	na	na	na
Funes et al. (2018) [42]	2010–2015	97/81	31/na	IIRC: B = 5 C = 8 D = 22 na = 62	na	IAC: 1° = 35 *IVC = 12* 2° = 62 *IVC = 62* *EBRT = 12*	IAC: 1° = 11 ^m^ 2° = 20	na	na	3 (10) ^n^	3 (100) ^n^	49 (12–72)	2 (2)	0 (0)	2 (2) ^o^

Legends: ^†^ = if available in italic are the treatments received prior IAC. Potential additional focal treatments are not mentioned; * = pre-enucleation treatments; HRF = histopathological risk factors for metastasis; na = not available; COG = Children’s Oncology Group; EBRT = external beam radiotherapy; IVC = intravenous chemotherapy; ICRB = International Classification for Retinoblastoma; RD = retinal detachment; RT = retinal tumor; SRS = subretinal seeds; VS = vitreous seeds; ON = optic nerve; MRI = magnetic resonance imagery; RE = Reese-Ellsworth Classification; TNM = Tumor Node Metastasis Staging; pTNM; pathological TNM; VH = vitreous hemorrhage; NVG = neovascular glaucoma; IIRC = International Intraocular Retinoblastoma Classification; ^a^ = estimated 0–6 month-interval between last treatment and SE; ^b^ = metastasis occurred 7 months after SE of a painful phthisic eye; ^c^ = one eye had suspicion of extraocular disease on histopathology; ^d^ = one patient treated for HRF in a non-IAC treated eye; ^e^ = one patient had SE elsewhere; ^f^ = two patients were lost to follow-up; ^g^ = three SE eyes after IAC2° had high-risk features (choroid and optic nerve invasion) and developed systemic metastasis despite receiving adjuvant chemotherapy. Two of them died; ^h^ = one SE eye had histopathological features defined as optic nerve invasion post lamina cribrosa, massive choroidal invasion and/or scleral invasion; ^i^ = a boy with retinoblastoma group D developed orbital recurrence 24 months after initial IAC followed by focal treatment, IVC and SE with pathology positive for NVG and ciliary body invasion. Subsequent work-up revealed metastatic disease. The patient is still alive without signs of metastases, 84 months after initial presentation. Mean time to metastatic disease from initial treatment was 26 months; ^j^ = pathological risk factors are not detailed in that study. All SE patients with HRF had undergone previous second line IAC. Two of them (one with histopathological anterior chamber and choroidal invasion and one with optic nerve invasion) died due to progressive disease despite multimodal treatment after enucleation; ^k^ = three patients treated with first line IAC developed metastases. All were successfully treated; ^l^ = one patient died of pinealoblastoma; ^m^ = 2 patients were treated with IAC until parents agreed to enucleate. One eye showed 1 massive choroid, the other had no histopathological HRF. Both patients are alive; ^n^ = 3 patients received adjuvant chemotherapy for histopathological high-risk factors (no further precision); ^o^ = 2 patients died of extraocular dissemination in the context of refusal of timely enucleation.

**Table 4:** Guidelines for secondary enucleation (SE) in the retinoblastoma treatment *.

	Definition	Clinical Situations	Recommendations
**Absolute indications for SE**	Conditions where maintaining an eye is considered to be life-threatening or associated with untreatable intraocular complications. Absolute indications are independent of the disease laterality	(1) Refractory disease despite salvage treatment (2) Direct or indirect sign(s) of disease exteriorization (3) Neovascular complications associated with untreatable ischemic retinopathy (4) Phthisis bulbi	- Suspicion of loss of tumor control should motivate enucleation within 7–14 days - Pre-enucleation MRI in case of loss of fundus view or suspicion of disease exteriorization - Histopathological analysis for evaluation of high-risk factors for metastasis should be performed in all SE cases
**Relative indications for SE**	Conditions where SE are may be indicated but where eye-preserving management could be considered, especially if it is the only seeing eye provided that certain criteria are met including: - parental full awareness of the residual risk of metastasis and death - guarantee that appropriate follow-up can be undertaken - availability of advanced diagnostic and therapeutic modalities in the treating centre	(1) Regressed tumor covering the optic nerve (2) Obscured tumor view secondary to poor pupillary dilatation, cataract, loss of corneal transparency and/or intravitreal haemorrhage (3) Tractional or rhegmatogenous retinal detachment (4) Neovascular complications (neovascular glaucoma, retinal and/or papillary neovascularization)	- In the case of a regressed tumor covering the optic nerve, patients should be followed with a 1.5 or 3 Tesla MRI, every 3 months for one year after treatment completion, then every 4 months for one year then every 6 months for at least one year. - Eyes with a regressed tumor covering the optic nerve after systemic chemoreduction may be consolidated with 2 courses of intra-arterial chemotherapy. - Eyes with ocular complications such as poor pupillary dilatation, cataract, tractional or rhegmatogenous retinal detachment, loss of corneal transparency, and/or intravitreal hemorrhage should also be followed with regular appropriate imaging until the fundus view is spontaneously or surgically restored. (*NB: Medical and surgical recommendations for the management of intraocular complications are beyond the scope of this article but should be given based on the available literature)*. - Histopathological analysis for evaluation of high-risk factors for metastasis should be performed in all SE cases.

* = the provided guidelines are the result of a consensus made on behalf of the European Retinoblastoma Group by retinoblastoma experts in ocular oncology, pediatric oncology and neuroradiology and do not reflect any evidence-based recommendations.

## Data Availability

Data sharing not applicable. No new data were created or analyzed in this study.

## Abbreviations

SE	secondary enucleation
MRI	Magnetic resonance imaging
OCT	optical coherence tomography
EURbG	European Retinoblastoma Group
OCT	optical coherence tomography
anti-VEGF	anti-vascular endothelial growth factor
